# Case report: Insulinomatosis: description of four sporadic cases and review of the literature

**DOI:** 10.3389/fendo.2023.1308662

**Published:** 2024-01-09

**Authors:** Delmar Muniz Lourenço, Maria Lucia Corrêa-Giannella, Sheila Aparecida Coelho Siqueira, Marcia Nery, Flavio Galvão Ribeiro, Elizangela Pereira de Souza Quedas, Manoel de Souza Rocha, Ramon Marcelino do Nascimento, Maria Adelaide Albergaria Pereira

**Affiliations:** ^1^ Unidade de Endocrinologia Genética (LIM-25), Hospital das Clínicas (HCFMUSP), Faculdade de Medicina, Universidade de São Paulo, São Paulo, Brazil; ^2^ Instituto do Câncer do Estado de São Paulo (ICESP), Faculdade de Medicina, Universidade de São Paulo, São Paulo, Brazil; ^3^ Laboratório de Carboidratos e Radioimunoensaio (LIM-18), Hospital das Clínicas (HCFMUSP), Faculdade de Medicina, Universidade de São Paulo, São Paulo, Brazil; ^4^ Divisão de Patologia, Hospital das Clínicas (HCFMUSP), Faculdade de Medicina, Universidade de São Paulo, São Paulo, Brazil; ^5^ Divisão de Endocrinologia e Metabologia, Hospital das Clínicas (HCFMUSP), Faculdade de Medicina, Universidade de São Paulo, São Paulo, Brazil; ^6^ Departamento de Radiologia, Hospital das Clínicas (HCFMUSP), Faculdade de Medicina, Universidade de São Paulo, São Paulo, Brazil

**Keywords:** hyperinsulinemic hypoglycemia, insulinoma, insulinomatosis, *MAFA* gene, long-term outcome, postprandial hypoglycemia

## Abstract

The best-known etiologies of hyperinsulinemic hypoglycemia are insulinoma, non-insulinoma pancreatogenous hypoglycemic syndrome, autoimmune processes, and factitious hypoglycemia. In 2009, a disease not associated with classic genetic syndromes and characterized by the presence of multiple pancreatic lesions was described and named insulinomatosis. We present the clinical and pathologic features of four patients with the diagnosis of insulinomatosis, aggregated new clinical data, reviewed extensively the literature, and illustrated the nature and evolution of this recently recognized disease. One of our patients had isolated (without fasting hypoglycemia) postprandial hypoglycemia, an occurrence not previously reported in the literature. Furthermore, we reported the second case presenting malignant disease. All of them had persistent/recurrent hypoglycemia after the first surgery even with pathology confirming the presence of a positive insulin neuroendocrine tumor. In the literature review, 27 sporadic insulinomatosis cases were compiled. All of them had episodes of fasting hypoglycemia except one of our patients. Only two patients had malignant disease, and one of them was from our series. The suspicion of insulinomatosis can be raised before surgery in patients without genetic syndromes, with multiple tumors in the topographic investigation and in those who had persistent or recurrent hypoglycemia after surgical removal of one or more tumors. The definitive diagnosis is established by histology and immunohistochemistry and requires examination of the “macroscopically normal pancreas.” Our case series reinforces the marked predominance in women, the high frequency of recurrent hypoglycemia, and consequently, a definitive poor response to the usual surgical treatment.

## 1 Introduction

Spontaneous hypoglycemia in non-diabetic subjects can be occasionally difficult to assess. The initial approach should establish whether hypoglycemia is associated with excessive and inappropriate insulin production. Hyperinsulinemic hypoglycemia (HH) may be caused by insulinomas, non-insulinoma pancreatogenous hypoglycemic syndrome (NIPHS) that can be idiopathic or secondary to bariatric surgery, autoimmune processes (anti-insulin or anti-insulin receptor antibodies), and surreptitious administration of insulin or oral hypoglycemic agents (factitious hypoglycemia) (1–3).

The main etiology of HH is insulinoma, a neuroendocrine tumor originating from pancreatic beta cells that is usually small (1 to 1.5 cm), benign, and unique (4, 5). Due to these characteristics, its excision results in a permanent cure, and the recurrence of hypoglycemia is practically non-existent (5). In approximately 10% to 15% of cases, insulinoma is a malignant tumor, and thus, hypoglycemia can recur due to metastatic tumor disease (2, 5). Insulinomas may occur as part of multiple endocrine neoplasia type 1 (MEN1) syndrome and, more rarely, as part of von Hippel Lindau syndrome (VHL) or neurofibromatosis type 1 (NF1). In these contexts, pancreatic tumors are usually multiple, and the recurrence of tumors is common (6–8).

Insulinomatosis, a disease described in 2009 by Anlauf et al. (8), is characterized by the presence of multiple pancreatic lesions, both tumors and/or pretumor lesions, and it is not associated with classic genetic syndromes, such as MEN1, VHL, or NF1. Recurrence of hypoglycemia after surgery is frequent and probably results from the growth of smaller tumors or the development of new tumors from preexisting microlesions (8). In the present article, we report four cases of sporadic insulinomatosis and a review of the literature on this topic.

## 2 Methods

The laboratory diagnosis of HH was made by concomitant determination of the plasma glucose and serum insulin, C-peptide, and proinsulin during an episode of spontaneous hypoglycemia or provoked by prolonged fasting. During the fasting test, capillary measurements of blood glucose and ketonemia were performed every 30 to 60 min. The fast was interrupted when the patient had symptoms of hypoglycemia and capillary glucose ≤50 mg/dL or, if asymptomatic, when capillary glucose was between 45 and 50 mg/dL; it was also interrupted when capillary β-hydroxybutyrate concentrations were >1 mmol/L, as high values are indicative of hypoinsulinemia and make further fasting unnecessary (1, 5). A mixed meal test (MMT) was performed when patients had symptoms in the postprandial period; in this case, blood was collected for the determination of glucose and insulin, before and every 30 min for 5 h after the ingestion of a 400-calorie meal (64% of carbohydrates). The biochemical criteria for the diagnosis of HH were those recommended by the Endocrine Society (glucose ≤ 55 mg/dL, insulin ≥ 3 µUI/mL, C-peptide ≥ 0.6 ng/dL, proinsulin ≥ 5 pmol/mL, and negative sulfonylurea screen) (1). A β-hydroxybutyrate concentration ≤0.3 mmol/L was considered for the diagnosis of HH, as previously described in our series of insulinomas (5). For the topographic diagnosis of the tumor(s), pancreatic computed tomography (CT) or magnetic resonance imaging (MRI) and endoscopic ultrasonography (EUS) of the pancreas were used. In one case, we used ^68^Gallium-DOTATATE PET-CT scanning to investigate the primary tumor and, in another, for recurrent hypoglycemia. Intra-arterial calcium stimulation with hepatic venous sampling (ASVS) for localization of insulinoma was performed when radiological methods were not able to identify the tumor (1, 5, 9).

Glucose was determined by the hexokinase method, insulin and C-peptide by chemiluminometric assays, and proinsulin by an immunoassay. Capillary ketonemia was assessed by the determination of blood β-hydroxybutyrate using an electrochemical method (MedSense Optium Meter). Blood sulfonylurea screening was performed by high-performance liquid chromatography (HPLC)/tandem mass spectrometry (Quest Diagnostics Nichols Institute, San Juan Capistrano, California, United States).

Sanger sequencing was provided to investigate germline mutations covering the coding area and splicing sites of the *MEN1* and *MAFA* genes. The protocol for the sequencing of the *MEN1* gene was conducted as previously reported (10, 11). Amplicons of exon 1 of the *MAFA* gene were amplified by polymerase chain reaction (PCR). PCR was performed in a total volume of 25 µL containing 2 µL of genomic DNA (200 ng), nuclease-free water 6 µL, 12.5 µL of Go Taq Green Master Mix (Promega, São Paulo, Brazil), primer forward 1.0 µL, primer reverse 1.0 µL, and DMSO 2.5 µL. The PCR thermocycling conditions were as follows: 10 min at 95°C, followed by 35 cycles of 40 min at 95°C, 40 min at 55°C as annealing temperature, followed by 40 min at 72°C and 10 min of final extension at 72°C.

The classification of genetic variants was conducted following the recommendations of the American College of Medical Genetics and Genomics and the Association for Molecular Pathologists (ACMG-AMP) (12).

## 3 Case presentations

### 3.1 Case 1

**Figure 1:**
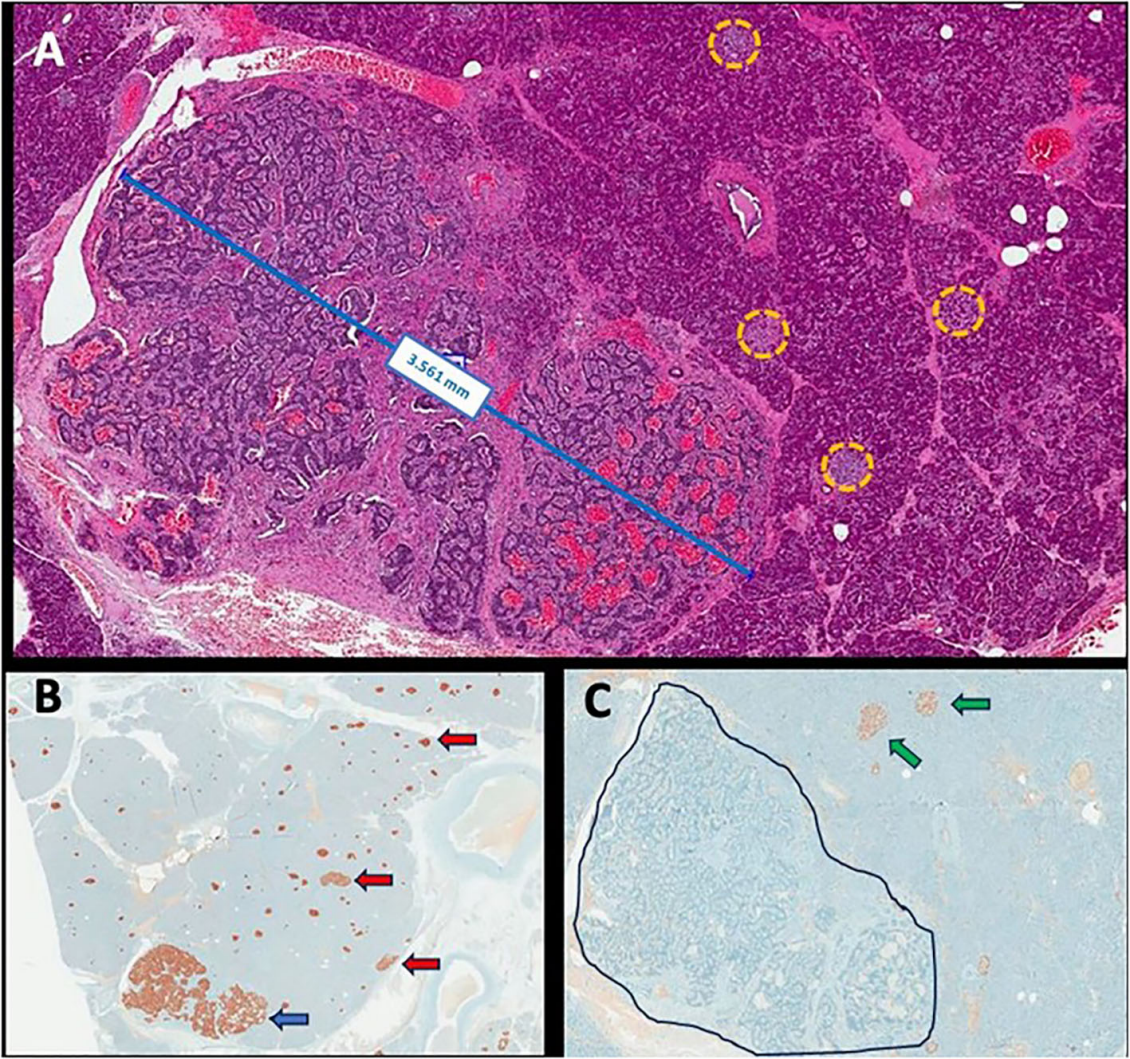
Representative histopathological images: neuroendocrine microtumor measuring 3.561 mm with several smaller clusters of neuroendocrine cells scattered within the macroscopically normal pancreas (yellow circles) (hematoxylin and eosin, ×200 magnification) **(A)**; immunohistochemistry for insulin showing positivity in neuroendocrine microtumor (blue arrow) and in the diverse smaller clusters of neuroendocrine cells (red arrow) (×100 magnification) **(B)**; immunohistochemistry for glucagon showing positivity in normal islets (green arrow) and negativity in the microtumor (delimited area) as in the clusters of neuroendocrine cells previously tested positive to insulin (×200 magnification) **(C)**.

A 52-year-old woman was admitted with a 1-year history of recurrent episodes of palpitations, sweating, and dizziness. She had no family history of hypoglycemia or diabetes. During one of these episodes, the laboratory workout showed glucose of 37 mg/dL, insulin of 13.4 µUI/mL, C-peptide of 2.7 ng/dL, negative ketonemia, and a negative sulfonylurea screen (**Table 1**). Abdominal MRI and EUS did not identify tumors, but ASVS revealed a 6.2 insulin gradient in the splenic artery, consistent with higher insulin production in the pancreatic body/tail. A second abdominal MRI showed a 1-cm T2-hyperintense lesion, previously unnoted in the pancreatic tail, which was enucleated, and the histological/immunohistochemical diagnosis was insulin-positive well-differentiated neuroendocrine tumor (Ki67 < 3%). There was no remission of the hypoglycemic episodes, and a second fasting test confirmed HH. An abdominal MRI, conducted after 3 months, showed two pancreatic tail lesions of 1.3 and 0.9 cm, the latter being located near the site of the previously enucleated tumor. The sequencing of the *MEN1* and *MAFA* genes was provided, but no mutation detected. The following variants, found in homozygosis and classified as benign by ACMG, were identified in MAFA gene: c.582T>C (p.HIS194=) (rs1872900), a synonymous missense variant and; c.221_223del (p.HIS208del (rs141816779), an in-frame variant. Distal pancreatectomy was performed, and histopathological examination revealed two tumors measuring 0.6 and 1.1 cm with several smaller ones (<0.5 cm) along the pancreatic body; all of them were well-differentiated tumors (Ki67 < 3%) with positive immunohistochemistry staining for insulin, chromogranin A, and synaptophysin and negative staining for glucagon, confirming the diagnosis of insulinomatosis (**Figure 1**). The patient remains free of hypoglycemia for 18 months after the second surgery (**Table 1**).

**Table 1:** Clinical and biochemical patterns of the four Brazilian cases with sporadic insulinomatosis.

Clinical and biochemical findings											
Case	Sex	Age^a^	Age at the last medical care	Follow-up time (years)	Fasting hypoglycemia	Symptoms	History of symptoms up to the hypoglycemia diagnosis (years)	Fasting test Biochemical/hormonal data^b^	Malignancy	Longer hypoglycemia-free time (months)	Recurrent hypoglycemia after last medical care
1	F	52	54	2	Yes	Palpitations Sweating Dizziness	1	Glucose = 37 mg/dL Insulin = 13.4 µUI/mL C-peptide = 2.7 ng/dL Ketonemia = negative SU = negative	No	18	No
2	F	49	61	12	No^c^	Sweating Palpitations Tremors (after meals or exercise) 4 kg weight gain	4	PFT (65 h) = negative MMT (60 min) = positive (**Figure 1**)	No	132	No
3	F	40	43	3	Yes	Disorientation Mental confusion Convulsive episodes during the dawn (2)	3 (months)	PFT (14 h) = positive Glucose = 42 mg/dL Insulin = 8.1 µUI/mL Proinsulin = 12.4 pmol/L C-peptide = 2.25 ng/dl β-HB < 0.1 mmol/L SU = negative	No	15	Yes
4	F	22	47	25	Yes	Sweating Palpitations Feelings of faintness (after periods of prolonged fasting) Tonic−clonic seizures (2)	1	Glucose = 26 mg/dL Insulin = 15 µUI/mL β-HB < 0.1 nmol/L (**Figure 2**)	Yes	60	Yes

PFT, prolonged fasting test; SU, sulfonylurea screen; MMT, mixed meal test; β-HB, β-hydroxybutyrate.

a Age at admission.

b Exams at admission.

c Postprandial hypoglycemia.

### 3.2 Case 2

**Figure 2:**
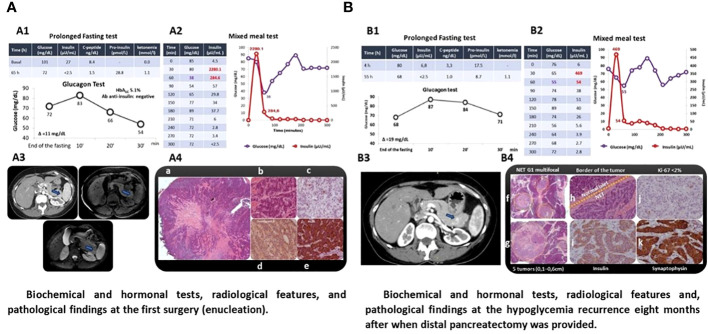
Patient with sporadic insulinomatosis (case 2) clinically presenting postprandial hyperinsulinemic hypoglycemia diagnosed for mixed meal test after normal response to prolonged fasting test. The same pattern of postprandial hypoglycemia was documented at the recurrence. Localization radiological exams and pathology documenting insulinomatosis after the first and second surgery are shown. **(A)** Biochemical and hormonal tests, radiological features, and pathological findings of the first surgery (enucleation). **(B)** Biochemical and hormonal tests, radiological features, and pathological findings of hypoglycemia recurrence 8 months after when distal pancreatectomy was provided. A1/B1. Prolonged fasting test was interrupted by the presence of ketonemia and the absence of hypoglycemia after 65 and 55 h with negative response at the glucagon test (increment <25 mg/dL); A2/B2. Mixed meal test was positive for hyperinsulinemic hypoglycemia 60 min on the first and second surgery; A3/B3. Pancreatic nodules were localized by computed tomography (A3/B3, arrow) and confirmed by endoscopic ultrasound (hypoechoic nodule measuring 1.5 cm in the pancreas tail (A3) and nodule in the distal extremity (1 cm) and hypervascularized nodule in the proximal segment of the pancreatic tail (0.6cm, arrow) (B3) whose biopsy was compatible with neuroendocrine neoplasia (IHC: insulin-positive); A4. Histology of the pancreatic nodule smaller (a) and larger in size (b) (hematoxylin and eosin); IHC documenting KI67 < 2 (c), positivity to chromogranin (d), and insulin (e); B4. Histology documenting three smaller pancreatic microtumors (f) and one larger tumor (g) stained by hematoxylin and eosin; transition between normal pancreatic tissue and neoplasia (h) (hematoxylin and eosin); IHQ revealing insulin-positive (i) well-differentiated neuroendocrine tumor (KI67 < 2%) (j), confirmed by positive IHC for synaptophysin (k).

A 49-year-old woman was admitted with a 4-year history of episodes of sweating, palpitations, and tremors after meals that were occasionally triggered by exercise. She did not present neuroglycopenic symptoms and gained 4 kg (**Table 1**). She had no family history of hypoglycemia or diabetes. After 65 h, a prolonged fasting test was interrupted when a β-hydroxybutyrate of 1.1 mmol/L was detected, concomitantly with glucose of 72 mg/dL, insulin of 2.5 µUI/mL, proinsulin of 28.8 pmol/L, and C-peptide of 1.5 ng/dL. An MMT showed HH at 60 min (glucose = 38 mg/dL and insulin = 284.5 µUI/mL) (**Figure 2A**; **Table 1**). An abdominal MRI detected a 1.5-cm tumor in the tail of the pancreas, and a pancreatic EUS showed a 1.4-cm hypoechoic nodule in the same location (**Figure 2A**). The patient underwent enucleation of the pancreatic tumor, and the histological diagnosis was a pancreatic neuroendocrine tumor with positive immunohistochemistry for insulin, chromogranin, and synaptophysin and a Ki67 labeling index <3% (grade 1 tumor) (**Figure 2A**). Eight months after the surgery, hypoglycemia recurred, and a second prolonged fasting test was performed; the test was interrupted after 55 h when hyperketonemia was detected (1.1 mmol/L) concomitantly with a glucose of 68 mg/dL, insulin of <2.5 µUI/mL, and C-peptide of 1 ng/dL (**Figure 2B**). An MMT showed reactive hypoglycemia at 60 min (glucose = 55 mg/dL and insulin = 54 µUI/mL) (**Figure 2B**). The sequencing of *MEN1* and *MAFA* genes did not show mutations.This patient harbored the same MAFA benign variants identified, in homozygosis, in case 1: c.582T>C (p.HIS194=) (rs1872900) and c.221_223del (p.HIS208del (rs141816779). An abdominal CT revealed two hypervascularized nodules in the pancreas, one of them measuring 1 cm at the extremity of the pancreatic tail and another measuring 0.6 cm between the tail and the body; the same lesions were identified in the EUS (**Figure 2B**). The patient underwent distal pancreatectomy that allowed the diagnosis of five neuroendocrine tumors measuring up to 0.6 cm (Ki67 < 3%) compatible with insulinomatosis (**Figure 2B**). She remains free of hypoglycemia to date, 11 years after the last surgery.

### 3.3 Case 3

A 40-year-old woman was admitted with a history of disorientation, mental confusion, and two convulsive episodes that occurred during the dawn. She had no family history of hypoglycemia or diabetes. A prolonged fasting test of 14 h showed glucose of 42 mg/dL, insulin of 8.1 µUI/mL, proinsulin of 12.4 pmol/L, C-peptide of 2.25 ng/dl, β-hydroxybutyrate of <0.1 mmol/L, and a negative sulfonylurea screen (**Table 1**). A pancreatic MRI and an EUS did not identify tumors, but a ^68^Gallium-DOTATATE PET-CT scanning revealed a focal area of uptake [standardized uptake value (SUV) of 7.1] in the head of the pancreas. Considering that the uptake could be physiological, the patient was submitted to an ASVS that, due to technical problems, was not able to localize the source of excessive insulin secretion. She received somatostatin analog monthly (30 mg) for 7 months with partial and insufficient improvement of hypoglycemia. Thus, the patient was referred to an exploratory laparotomy with intraoperative palpation and ultrasonography, which identified two lesions, one 0.6 cm in the body of the pancreas and another smaller one in the pancreatic tail; central pancreatectomy and resection of the distal nodule were performed, and the histological analysis revealed multiple well-differentiated neuroendocrine microtumors (Ki67 < 3%) measuring up to 0.5 cm compatible with insulinomatosis. The patient remained free of hypoglycemia for 15 months when tests performed after 12 h of fasting showed glucose of 50 mg/dL, insulin of 12.2 µUI/mL, and C-peptide of 3.64 ng/dL. She is now undergoing radiological examination for the topographical diagnosis. There was no mutation found in *MEN1* and *MAFA* genes. This patient harbored the same *MAFA* benign variants identified, in homozygosis, in cases 1 and 2: c.582T>C (p.HIS194=) (rs1872900) and c.221_223del (p.HIS208del (rs141816779).

### 3.4 Case 4

**Figure 3:**
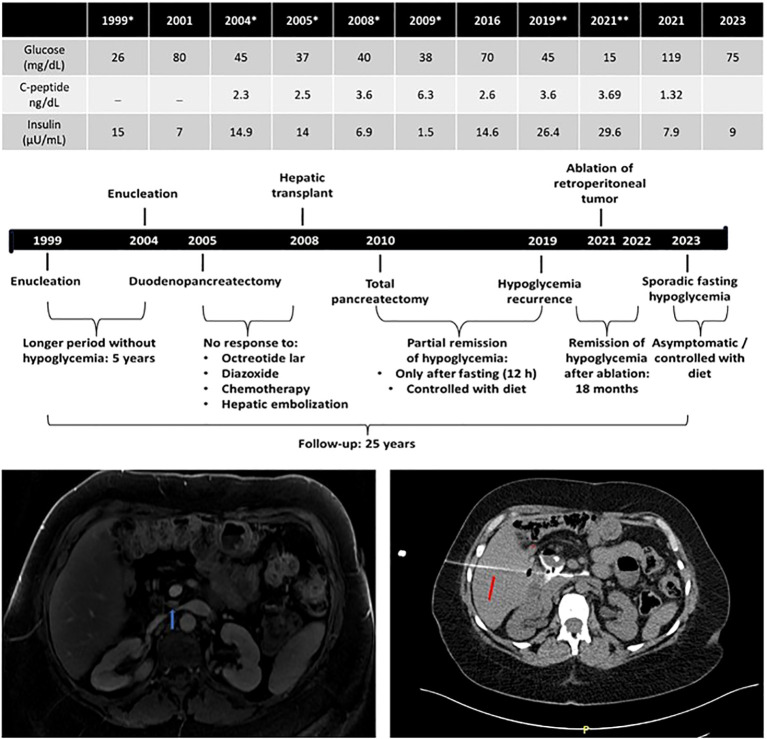
Long-term outcome (25 years) of a patient (case 4) diagnosed with malignant insulinomatosis 7 years after multiple surgical treatments by recurrent/persistent hypoglycemia. *, basal biochemical/hormonal values documenting hyperinsulinemic hypoglycemia before surgical procedures: 1999, enucleation; 2004, enucleation; 2005; subtotal duodenopancreatectomy; 2008, liver transplant after biopsy confirming hepatic metastasis and unsuccessful multiple treatments (diazoxide, somatostatin analogs, chemotherapy, and hepatic embolization); 2019/2021, basal biochemical/hormonal values documenting hyperinsulinemic hypoglycemia before radiofrequency ablation (red arrow: needle of ablation) of retroperitoneal tumoral mass (blue arrow) detected by magnetic resonance imaging in 2021.

A 22-year-old woman was admitted with a 1-year history of episodes of sweating, palpitations, and feelings of faintness after periods of prolonged fasting; on two occasions, she reported tonic−clonic seizures. She had no family history of hypoglycemia or diabetes. During one of the episodes, the following measurements were recorded: glucose of 26 mg/dL and insulin of 15 µUI/mL (**Table 1**). An abdominal MRI showed a 1.4-cm tumor between the head and body of the pancreas. The patient was referred for tumor enucleation, and histological examination showed a grade 1 pancreatic neuroendocrine tumor with positive immunohistochemistry for chromogranin and insulin and a Ki67 labeling index <3% (1999). The patient remained asymptomatic and had normal biochemical and hormonal findings for 5 years, after which she presented recurrence of hypoglycemia. An HH was redocumented, MRI topographic exploration was negative, and an ASVS showed hypersecretion of insulin from the head of the pancreas. The patient was referred for a second surgery which allowed the identification and enucleation of a small tumor (0.5 cm), whose pathology revealed a neuroendocrine tumor with a Ki67 <5%, but remission of hypoglycemia was not achieved (2004). Again, topographic investigation remained negative by MRI and positive by ASVS at the same localization. With this, pancreatoduodenectomy was performed (2005). The histological diagnosis was compatible with insulinomatosis by the presence of multiple neuroendocrine tumors (up to 0.7 cm), with Ki67 between 2% and 10%. Also, the sequencing of *MEN1* and *MAFA* genes did not reveal any mutations. This patient harbored the same *MAFA* benign variants identified, in homozygosis, in cases 1, 2 and 3: c.582T>C (p.HIS194=) (rs1872900) and c.221_223del (p.HIS208del (rs141816779). Due to persistent hypoglycemia, a new radiological exam was done (^111^In-Octreotide scintigraphy and abdominal MRI) and suggested hepatic metastasis. A hepatic biopsy confirmed the diagnosis of metastatic neuroendocrine neoplasia with positive immunohistochemistry for insulin (2005). Clinical treatment (diazoxide and octreotide) was initiated with partial and transient improvement of hypoglycemia. Hepatic artery embolization and systemic chemotherapy were performed without hypoglycemia remission. Between 32 and 34 years of age, as the patient had metastasis only in the liver and, probably, a persistent pancreatic disease, she was referred for a liver transplant (2008) with transient remission of hypoglycemia and then underwent total pancreatectomy (2010); histopathological and immunohistochemistry examination revealed metastatic neuroendocrine neoplasia and insulinomatosis, respectively. Transient improvement of hypoglycemia after total pancreatectomy, without development of diabetes, was followed by gradual and progressive worsening requiring fractionated diet in subsequent years (2011–2020), but abdominal MRI exams performed periodically and, sometimes, complemented with ^111^In-Octreotide scintigraphy or ^68^Gallium-DOTATATE PET-CT, were persistently negative. Between 2005 and 2021, the somatostatin analog was offered to the patient for short periods of less than 6 months immediately after failure to remit hypoglycemia for different treatments such as liver embolization, liver transplant, or total pancreatectomy. However, intolerance or unsatisfactory adherence prevented an accurate assessment of the potential of this drug to correct hypoglycemia in this patient. At 45 years old, after an episode of neuroglycopenia (glucose of 15 mg/dL, insulin of 29.6 µUI/mL, C-peptide of 3.69 ng/dL, and β-hydroxybutyrate of <0.1 mmol/L), an abdominal MRI identified a small suspect lesion near the site where it would be the head of the pancreas, whereas a ^68^Gallium-DOTATATE PET-CT scan was inconclusive (2021). The patient was referred for radiofrequency ablation guided by CT. After 1 h, glucose measurements were approximately 150 and 200 mg/dL, and during the next 6 months, she required exogenous insulin for treating secondary diabetes mellitus. Since then, she began to experience new episodes of fasting hypoglycemia remitting after a meal, which led to the suspension of exogenous insulin. The pre- and postprandial plasma glucose values are approximately 90 and 160 mg/dL, respectively (**Figure 3**).

## 4 Discussion

Insulinomatosis is a pancreatic disease characterized by multiple micro- and macrotumors and foci of beta-cell hyperplasia (8, 13–15). Although there were previous reports of “multiple” insulinomas (13), the pathological bases that allowed its differentiation from insulinoma were only defined in 2009 by Anlauf et al., who evaluated the histological and immunohistochemical characteristics of the tumors of the pancreas in 253 patients with sporadic single insulinoma, 13 patients with insulinoma associated with MEN1, and 14 patients (mean age of 41 ± 12 years old; 10 women and 4 men) with what these authors called insulinomatosis (8). In this condition, the histological evaluation of the pancreas revealed several insulinomas [microtumors (<5 mm) and macrotumors (≥5 mm)] and multiple conglomerates of beta cells of variable size called insulin-expressing monohormonal endocrine cell clusters (IMECCs). Immunohistochemistry of tumors and IMECCs was positive for insulin and negative for glucagon. IMECCs were initially considered precursor lesions of micro- and macrotumors, and Anlauf et al. highlighted that the presence of these pretumoral lesions only occurred in insulinomatosis and never in sporadic insulinomas or associated with MEN1. Recently, the term IMECC was incorporated as microtumors (<0.5 cm). Immunohistochemistry in sporadic benign insulinoma, which in the authors’ experience was always a single tumor, was positive for insulin and negative for glucagon. In the tumors of patients with MEN1, immunohistochemistry was variable in the different tumors and could be negative or positive for insulin, glucagon, or pancreatic polypeptide (PP). Thus, in this genetic syndrome, tumors may be non-functioning, or they may express one of the islet hormones. It was notable that patients with insulinomatosis showed a high rate of HH persistence/recurrence despite repeated surgeries. Insulinomatosis was a benign disease in all patients except one who presented with metastatic disease (8). After this description, others emerged describing new cases, all highlighting that, in insulinomatosis, the recurrence rate of hypoglycemia after surgery is high (16–19), contrasting with the absence of recurrent hypoglycemia in sporadic well-differentiated insulinomas (8) and with a low recurrence rate of hypoglycemia, even with high rates of tumoral recurrence, in cases with MEN1-related insulinoma undergoing pancreatic surgery (~7%) (6–8, 20, 21).

We described four adult women presenting HH with histological and immunohistochemical diagnoses of insulinomatosis. All of them had biochemical persistence or recurrence of hypoglycemia after the initial pancreatic surgery. One of the patients (case 1) had persistent hypoglycemia, while the second (case 2) had recurrent hypoglycemia 8 months after enucleation of what was thought to be sporadic insulinomas, and they were subsequently submitted to partial pancreatectomy, which allowed the histological diagnosis of insulinomatosis. They remained free of hypoglycemia 1.5 and 11 years, respectively, after the last procedure. Case 3 had persistent HH after partial pancreatectomy, which allowed the diagnosis of insulinomatosis; this patient had recurrent HH 15 months after the last surgery. In all these three cases, the return of hypoglycemia after surgery is linked to the recurrence of pancreatic disease (micro- and macrotumors). In case 4, the only one with a diagnosis of metastatic malignant insulinomatosis, recurrences of hypoglycemia over a period of 25 years of follow-up were due to the growth of new tumors in the pancreas, but also due to extrapancreatic (metastatic) disease. It is interesting to highlight that this is the only patient in the literature who underwent a liver transplant and subsequent radiofrequency ablation of the residual retroperitoneal lesion (**Figure 3**).

Overall, there are 27 cases documented with sporadic insulinomatosis including our four cases presently reported (8, 17, 22–25) (**Table 2**). Of note, one of these 27 cases previously reported had proinsulinomatosis, and this diagnosis was probable in another sporadic case with undetectable insulin and high levels of proinsulin (8, 21, 23). The marked predominance in women is reinforced by the present cases (81%; 22/27). The mean age at the diagnosis of hypoglycemia in these sporadic cases was 42 ± 13.4 years (17–64) with 41% of them (11/27) undergoing two or more surgical resections (2 ± 1.06; 1–5) for recurrent hypoglycemia (**Table 2**). All of our cases had at least one recurrence and three of them underwent more than one surgery. The recurrence was pancreatic and due to benign insulinomatosis in three of them, while case 4 had recurrence secondary to pancreatic insulinomatosis and to metastatic insulinomatosis. This is the second case with sporadic malignant insulinomatosis reported so far (7.4%; 2/27) (**Tables 1**, **2**). The first one, reported by Anluf et al. (8), underwent wedge resection for hepatic metastasis. This predominance of benign behavior is also observed in familial insulinomatosis, as malignancy was not present in any of the 12 cases of insulinomatosis from three families reported so far (8, 13, 14, 22, 26).

**Table 2:** Clinical data of all 27 cases with sporadic insulinomatosis reported by literature, including four patients of the present study.

Reference	Case	Sex	Age^a^	Recurrent hypoglycemia after first surgery	Number of surgeries	Biochemical/hormonal data	Fasting hypoglycemia	Malignancy	Proinsulinomatosis	Other oncologic treatments	^68^Ga-DOTATATE PET/CT	First surgery	Last surgery
Anluf (2009) (8)	1	F	17	Yes	2	NA	Yes	No	No		–	PP	Enucleation
	2	F	26	No	1	NA	Yes	No	No		–	PP	–
	3	F	28	Yes	3	NA	Yes	No	No		–	Enucleation	PP
	4	F	32	Yes	2	NA	Yes	No	No		–	Enucleation	PP + enucleation
	5	F	37	No	1	NA	Yes	No	No		–	PP	–
	6	F	40	Yes	5	NA	Yes	Yes	No		–	Enucleation	Lymphadenectomy + Wedge resection (liver)
	7	F	43	No	1	NA	Yes	No	No		–	PP	–
	8	F	45	No	1	NA	Yes	No	No		–	PP	–
	9	F	49	No	1	NA	Yes	No	No		–	PP	–
	10	M	50	No	1	NA	Yes	No	No		–	PP	–
	11	M	57	No	1	NA	Yes	No	No		–	PP	No
	12	M	56	Yes	1	NA	Yes	No	No		–	PP	– (autopsy)
	13	M	59	NA	1	NA	Yes	No	No		–	PP	–
Iacovazzo (22)^c^	14	F	17	Yes	3	Yes	Yes	No	No		–	Enucleation	Whipple
	15	F	48	Yes	1	Yes	Yes	No	No	Octreotide^b^	–	PP	–
	16	F	64	Yes	2	Yes	Yes	No	Yes		–	PP	PP
	17	F	47	No	1	Yes	Yes	No	No		–	PP	–
	18	F	51	No	1	Yes	Yes	No	No		–	PP	–
	19	F	20	NA	1	No	Yes	No	No		–	PP	–
Snaith (2020)^d^ (17)^d^	20	F	40	Yes	3	Yes	Yes	No	No	Octreotide^b^ Everolimus^b^	Negative^e^	Enucleation	TP
Mintziras (2021)^d^ (23)	21	F	48	Yes	2	Yes	Yes	No	Yes		Positive	Enucleation	PP
Anoshkin (2021) (24)	22	M	60	No	1	No	Yes	No	No		Negative^e^	PP	–
Tartaglia (2022) (25)	23	F	41	Yes	1	Yes	Yes	No	No	Octreotide^f^	Positive	PP	–
Lourenço (2023)^d^	24	F	52	Yes	2	Yes	Yes	No	No		–	Enucleation	PP
	25	F	49	Yes	2	Yes	No^g^	No	No		–	Enucleation	PP
	26	F	40	Yes	1	Yes	Yes	No	No	Octreotide^b^	Inconclusive	PP	–
	27	F	22	Yes	6	Yes	Yes	Yes	No	Octreotide^b^	Inconclusive^e^	Enucleation	TP Hepatic transplant RFA

PP, partial pancreatectomy; TP, total pancreatectomy; RFA, radiofrequency ablation.

a Age at diagnosis.

b Poor or partial responses or intolerance to somatostatin analogs or everolimus.

c Nine sporadic cases were genetically investigated by Iacovazzo et al. (three of them had been clinically reported by Anluf et al. (8), and they were negative for germline and somatic MAFA mutations.

d Six additional cases whose genetic testing was negative for germline MAFA mutations.

e Performed during follow-up in one of the episodes of hypoglycemia recurrence after the first surgery.

f Highly responsive to a somatostatin analog.

g Positive mixed meal test.

NA, not available.

It is not possible to differentiate between insulinoma and insulinomatosis before surgery. Our previous experience with insulinoma (5) allows us to make some comparisons between isolated insulinoma and insulinomatosis. All patients with insulinomatosis were women, while an even sex distribution was observed in patients with insulinoma; however, although the absolute prevalence of insulinomatosis in women should be emphasized, this does not allow the differentiation between the two conditions (1, 5, 8). The clinical picture is not different in patients with insulinomatosis and insulinoma, but we would like to point out that one of the four patients with insulinomatosis had hypoglycemia only in the postprandial period, with no fasting hypoglycemia, while this did not occur in any of our patients with isolated insulinoma (5). From our knowledge, this is the first case with insulinomatosis presenting postprandial hypoglycemia documented with mixed meal test reported so far (**Figure 2**), contrasting with fasting hypoglycemia noticed in the other 26 sporadic (**Table 2**) (8, 17, 22–25) and in 11 familial cases with insulinomatosis (8, 13, 22, 26).

The biochemical evaluation is identical in insulinoma and insulinomatosis, and the findings of topographic investigation may also be similar, especially when only one tumor is identified. In insulinomatosis, the multiple tumors can be synchronous or asynchronous, and in the latter case, one tumor may appear before the other(s) develop. Failure to identify tumors by the usual radiological methods (magnetic resonance imaging, computed tomography), including ^68^Gallium-DOTATATE PET-CT and even endoscopic ultrasound, is common as most tumors in insulinomatosis are too small (microtumors, <5 mm) to be detected by these imaging diagnostic techniques, making preoperative diagnosis of the disease difficult. This context should motivate the performance of an ASVS to localize the source of excessive insulin secretion to guide surgical removal (1). It is important to highlight that ASVS may have the same limitations as radiological methods due to the simultaneous and additive functionality of the multiple microtumors spread across the pancreas, without the identification of a dominant area responsible for hypersecretion of insulin. Only the identification of more than one territory as a source of insulin hypersecretion can raise the suspicion of insulinomatosis. Therefore, the presurgical diagnosis of this disease can only be considered when several tumors are identified or suspected by the usual radiological methods or by ASVS. When multiple tumors are identified, it is mandatory to exclude clinically and/or molecularly the diagnosis of MEN1 (6–8).

Thus, the definitive diagnosis of insulinomatosis is histological and requires examination of the peritumoral “macroscopically normal pancreas,” not just the tumor. In the “normal” pancreas, micro- and macrotumors will be identified (8, 27, 28). If the surgical approach was tumor enucleation, a complete analysis of the pancreas is not feasible. In this case, the patient may remain symptomatic because there are other macro- or microtumors not identified by presurgical (MRI/CT or EUS) or intrasurgical methods. Hypoglycemia may also improve and recur later due to the growth of preexisting tumors or the development of new ones. The histological differential diagnosis between insulinomatosis and MEN1 is easily established when the criteria mentioned above are adopted (8, 18). In patients with MEN1, the occurrence of multiple neuroendocrine tumors is frequent, but in general, immunohistochemistry shows that they are quite diverse and may be non-functioning or may produce insulin, glucagon, or pancreatic polypeptide. In insulinomatosis, micro- and/or macrotumors are present, and immunohistochemistry is quite monotonous and always positive only for insulin in the various existing lesions. Tumor recurrence occurs earlier in patients with insulinomatosis than in those with MEN1 (8). In our service, we did not have any recurrence of hypoglycemia in patients operated on for benign insulinoma or insulinoma with MEN1 (5).

Insulinomatosis is a disease that requires a difficult and, sometimes, exhausting surgical treatment. Due to the rarity of the disease, the treatment with somatostatin analogs has not yet been evaluated. In our series, one of our cases (case 3) had no response after a short period of drug use, while another did not tolerate the analog (case 4) (**Table 2**). Also, two cases with sporadic insulinomatosis and three with familial insulinomatosis received this treatment, and response/tolerance seemed to be unsatisfactory (14, 17, 22) (**Table 2**). However, one clinical finding that drew our attention was the good response to therapy with a somatostatin analog in a patient with suspected insulinomatosis. This diagnosis assumption was made because the patient had HH, two pancreatic tumors, and negative clinical and genetic findings for MEN1 (data not shown). She was not included in this series because she did not undergo surgery and, therefore, did not have a definitive histological diagnosis of insulinomatosis. This patient showed absolute remission of hypoglycemia and a decrease in tumor size after therapy with the analog. A similar response was recently described in a patient with insulinomatosis (25) (**Table 2**).

In this scenario, the role of somatostatin analogs as one of the options to control hypoglycemia and the growth of tumors in insulinomatosis cases requires additional studies to elucidate if it diverges with the poor response noticed with most of the isolated insulinomas (5). Anyway, a possible explanation for this potential difference could be that the production and secretion of insulin, as well as the growth of micro- and macrotumors, can be more easily controlled by beta-cell inhibitors than by an autonomous tumor. In this rationale, it is relevant to consider that there are only a few cases investigated with ^68^Gallium-DOTATATE PET-CT, an exam that could predict response to somatostatin analogs (**Table 2**). In fact, only three sporadic cases, including case 3 of the present study, had ^68^Gallium-DOTATATE PET-CT as an initial approach. This exam was negative in our case 3, who had no clinical response to the analog, while it was positive in that patient highly responsive to somatostatin analogs and in another with proinsulinomatosis (23, 25). Other five cases had ^68^Gallium-DOTATATE PET-CT during the investigation of hypoglycemia recurrence (two familial, three sporadic), including our case 4, and in all of them, this exam was negative or inconclusive to localization of tumors (17, 22, 24). We consider that, in any case with this disease, we must attempt clinical treatment with the analog before performing partial or total pancreatectomy. In fact, total pancreatectomy is not always a definitive solution to hypoglycemia in insulinomatosis, as documented in our case 4 and as previously reported by Snaith et al. (17).

Insulinomatosis is generally sporadic, but familial cases with autosomal dominant inheritance have been described (8, 13, 22, 26). Although a family with patients affected by multiple insulinomas and hypoglycemia had been reported in 1977 (13), only in 2018 were germline mutations in the *MAFA* gene identified in two families in which members with hypoglycemia secondary to insulinomatosis coexisted with members diagnosed with diabetes mellitus (22). An important issue is how the same genetic alteration can lead to apparently opposite diseases (22). More recently, a novel missense *MAFA* mutation was reported in a kindred with familial insulinomatosis (26). Overall, a germline *MAFA* mutation has been documented in only three families to date and one of them was Brazilian (22, 26). Of note, our unrelated sporadic cases do not harbor the mutation p.Ser64Phe found in large Brazilian family with insulinomatosis previously reported (22). Overall, germline *MAFA* mutations were not found in our 4 cases and 11 other sporadic cases (17, 22, 23). By negative genetic analysis, the possibility of familial insulinomatosis as a result of a *de-novo MAFA* mutation or incomplete penetrance or even a founder mutation from apparently unrelated clusters was excluded in our cases.

It is noteworthy that the same in-frame deletion was found in all four currently reported cases with proven insulinomatosis and in a fifth case with presumed insulinomatosis (not yet operated on due to excellent response to somatostatin analogue). This variant, located in a region with ten successive histidine repeats, was previously documented in a case of proinsulinomatosis and its benign nature is reinforced by its very high frequency in genomic banks (>81% in genomes from gnomD) (14, 23).

In addition, in nine sporadic cases, somatic *MAFA* mutations were excluded (22). While such mutations have not been identified so far, we cannot entirely rule out, due to the rarity of the disease, whether they can play a role in the pathogenesis of sporadic insulinomatosis. Recently, new copy number variations in *ATRX*, *FOXL2*, *IRS2*, and *CEBPA* genes were documented in macro- and microtumors of one case with sporadic insulinomatosis, suggesting a potential role of these genes in the pathogenesis of this rare disorder (24).

## 5 Conclusions

The main considerations to be drawn from the analysis of our data and those of the literature are that the suspicion of insulinomatosis can be raised: 1) before surgery, in patients with multiple tumors in the topographic investigation (after MEN1 exclusion) or in whom more than one territory was identified as sources of insulin hypersecretion by ASVS; 2) after surgical removal of one or more tumors, in those patients who had no improvement or recurrence of the hypoglycemic condition; and 3) the definitive diagnosis is established by histology and immunohistochemistry and requires examination of the “macroscopically normal pancreas.”

## Data availability statement

The original contributions presented in the study are included in the article/supplementary material. Further inquiries can be directed to the corresponding author.

## Ethics statement

The studies involving humans were approved by CAPPesq (no. 70989723.0.0000.0068). The studies were conducted in accordance with the local legislation and institutional requirements. The participants provided their written informed consent to participate in this study. Written informed consent was obtained from the individual(s) for the publication of any potentially identifiable images or data included in this article.

## Author contributions

DL: Data curation, Formal analysis, Funding acquisition, Investigation, Methodology, Visualization, Writing – original draft, Writing – review & editing. MC-G: Data curation, Formal analysis, Funding acquisition, Writing – original draft, Writing – review & editing. SS: Data curation, Formal analysis, Methodology, Writing – review & editing. MN: Data curation, Formal analysis, Writing – review & editing. FR: Formal analysis, Investigation, Writing – review & editing. EQ: Formal analysis, Investigation, Methodology, Writing – review & editing. MR: Data curation, Formal analysis, Investigation, Methodology, Writing – review & editing. RN: Data curation, Formal analysis, Investigation, Writing – review & editing. MP: Conceptualization, Data curation, Formal analysis, Investigation, Methodology, Project administration, Supervision, Visualization, Writing – original draft, Writing – review & editing.

## References

[1] Cryer PE Axelrod L Grossman AB Heller SR Montori VM Seaquist ER. Evaluation and management of adult hypoglycemic disorders: an Endocrine Society Clinical Practice Guideline. J Clin Endocrinol Metab (2009) 94(3):709–28. doi: 10.1210/jc.2008-1410 19088155

[2] Woo C-Y Jeong JY Jang JE Leem J Jung CH Koh EH. Clinical features and causes of endogenous hyperinsulinemic hypoglycemia in Korea. Diabetes Metab J (2015) 39(2):126–31. doi: 10.4093/dmj.2015.39.2.126 PMC4411543 25922806

[3] Yamada Y Kitayama K Oyachi M Higuchi S Kawakita R Kanamori Y. Nationwide survey of endogenous hyperinsulinemic hypoglycemia in Japan (2017-2018): Congenital hyperinsulinism, insulinoma, noninsulinoma pancreatogenous hypoglycemia syndrome and insulin autoimmune syndrome (Hirata's disease). J Diabetes Investig (2020) 11(3):554–63. doi: 10.1111/jdi.13180 PMC7232294 31742894

[4] Grant CS. Insulinoma. Best Pract Res Clin Gastroenterol (2005) 19(5):783–98. doi: 10.1016/j.bpg.2005.05.008 16253900

[5] Camara-de-Souza AB Toyoshima MTK Giannella ML Freire DS Camacho CP Lourenço DM Jr.. Insulinoma: A retrospective study analyzing the differences between benign and Malignant tumors. Pancreatology (2018) 18(3):298–303. doi: 10.1016/j.pan.2018.01.009 29452754

[6] Tonelli F Giudici F Giusti F Brandi ML. Gastroenteropancreatic neuroendocrine tumors in multiple endocrine neoplasia type 1. Cancers (Basel). (2012) 4(2):504–22. doi: 10.3390/cancers4020504 PMC3712700 24213321

[7] Tonelli F Giudici F Nesi G Batignani G Brandi ML. Operation for insulinomas in multiple endocrine neoplasia type 1: When pancreatoduodenectomy is appropriate. Surgery (2017) 161(3):727–34. doi: 10.1016/j.surg.2016.09.017 27863775

[8] Anlauf M Bauersfeld J Raffel A Koch CA Henopp T Alkatout I. Insulinomatosis: a multicentric insulinoma disease that frequently causes early recurrent hyperinsulinemic hypoglycemia. Am J Surg Pathol (2009) 33(3):339–46. doi: 10.1097/PAS.0b013e3181874eca 19011561

[9] Thompson SM Vella A Thompson GB Rumilla KM Service FJ Grant CS. Selective arterial calcium stimulation with hepatic venous sampling differentiates insulinoma from nesidioblastosis. J Clin Endocrinol Metab (2015) 100(11):4189–97. doi: 10.1210/jc.2015-2404 PMC4702445 26312578

[10] Toledo RA Lourenço DM Jr Coutinho FL Quedas E Mackowiack I MaChado MC. Novel MEN1 germline mutations in Brazilian families with multiple endocrine neoplasia type 1. Clin Endocrinol (Oxf). (2007) 67(3):377–84. doi: 10.1111/j.1365-2265.2007.02895.x 17555499

[11] Lourenço DM Jr Toledo RA Coutinho FL Margarido LC Siqueira SA dos Santos MA. The impact of clinical and genetic screenings on the management of the multiple endocrine neoplasia type 1. Clinics (Sao Paulo). (2007) 62(4):465–76. doi: 10.1590/S1807-59322007000400014 17823710

[12] Richards S Aziz N Bale S Bick D Das S Gastier-Foster J. Standards and guidelines for the interpretation of sequence variants: a joint consensus recommendation of the American College of Medical Genetics and Genomics and the Association for Molecular Pathology. Genet Med (2015) 17(5):405–24. doi: 10.1038/gim.2015.30 PMC4544753 25741868

[13] Tragl KH Mayr WR. Familial islet-cell adenomatosis. Lancet (1977) 2(8035):426–8. doi: 10.1016/s0140-6736(77)90609-2 70643

[14] Christ E Iacovazzo D Korbonits M Perren A. Insulinomatosis: new aspects. Endocr Relat Cancer. (2023) 30(6):e220327. doi: 10.1530/ERC-22-0327 36952647

[15] de Herder WW Klöppel G. One hundred years after the discovery of insulin and glucagon: the history of tumors and hyperplasias that hypersecrete these hormones. Endocr Relat Cancer. (2023) 30(9):e230046. doi: 10.1530/ERC-23-0046 37310813

[16] Jaramillo Chacón H González Devia D López Panqueva RP Cañon Solano D Aguirre Matallana D Rey Rubiano AM. Insulinomatosis: a very rare cause of pancreatic neuroendocrine tumor. Rev Colombiana Endocrinología Diabetes Metabolismo. (2020) 7(2):76–85. doi: 10.53853/encr.7.2.607

[17] Snaith JR McLeod D Richardson A Chipps D. Multifocal insulinoma secondary to insulinomatosis: persistent hypoglycemia despite total pancreatectomy. Endocrinol Diabetes Metab Case Rep (2020) 20:91. doi: 10.1530/EDM-20-0091 PMC7849475 33434149

[18] Guojing Y Jingtao D Li Z Yiming M. A special form of pancreatic hyperinsulinemic hypoglycemia - insulinomatosis: A case report. Endocrine Rev (2014) 35 Suppl 1:i1–1153. doi: 10.1093/edrv/35.supp.1

[19] Jenni S Antwi K Fani M Wild D Heye T Gloor B. Multifocal insulinomas (insulinomatosis) in GLP-1-receptor PET/CT. Endocrine Abstracts. (2015) 37:EP1115. doi: 10.1530/endoabs.37.EP1115

[20] van Beek DJ Nell S Verkooijen HM Borel Rinkes IHM Valk GD (on behalf of the DutchMEN study group). Surgery for multiple endocrine neoplasia type 1-related insulinoma: long-term outcomes in a large international cohort. Br J Surg (2020) 107(11):1489–99. doi: 10.1002/bjs.11632 PMC7540387 32352164

[21] Gonçalves TD Toledo RA Sekiya T Matuguma SE Maluf Filho F Rocha MS. Penetrance of functioning and nonfunctioning pancreatic neuroendocrine tumors in multiple endocrine neoplasia type 1 in the second decade of life. J Clin Endocrinol Metab (2014) 99(1):E89–96. doi: 10.1210/jc.2013-1768 24178797

[22] Iacovazzo D Flanagan SE Walker E Quezado R de Sousa Barros FA Caswell R. MAFA missense mutation causes familial insulinomatosis and diabetes mellitus. Proc Natl Acad Sci U S A. (2018) 115(5):1027–32. doi: 10.1073/pnas.1712262115 PMC5798333 29339498

[23] Mintziras I Peer K Goerlach J Goebel JN Ramaswamy A Slater EP. Adult proinsulinomatosis associated with a MAFA germline mutation as a rare cause of recurrent hypoglycemia. Pancreas (2021) 50(10):1450–3. doi: 10.1097/MPA.0000000000001933 35041347

[24] Anoshkin K Vasilyev I Karandasheva K Shugay M Kudryavtseva V Egorov A. New regions with molecular alterations in a rare case of insulinomatosis: case report with literature review. Front Endocrinol (Lausanne). (2021) 12:760154. doi: 10.3389/fendo.2021.760154 34737724 PMC8563021

[25] Tartaglia A Busonero G Gagliardi L Boddi V Pieri F Nizzoli M. Complete remission of recurrent multiple insulin-producing neuroendocrine tumors of the pancreas with somatostatin analogs: a case report and literature review. Discovery Oncol (2022) 13(1):66. doi: 10.1007/s12672-022-00531-z PMC9287506 35838801

[26] Fottner C Sollfrank S Ghiasi M Adenaeuer A Musholt T SChad A. Second MAFA variant causing a phosphorylation defect in the transactivation domain and familial insulinomatosis. Cancers (Basel). (2022) 14(7):1798. doi: 10.3390/cancers14071798 35406570 PMC8997416

[27] Anlauf M Schlenger R Perren A Bauersfeld J Koch CA Dralle H. Microadenomatosis of the endocrine pancreas in patients with and without the multiple endocrine neoplasia type 1 syndrome. Am J Surg Pathol (2006) 30(5):560–74. doi: 10.1097/01.pas.0000194044.01104.25 16699310

[28] Klöppel G Anlauf M Perren A Sipos B. Hyperplasia to neoplasia sequence of duodenal and pancreatic neuroendocrine diseases and pseudohyperplasia of the PP-cells in the pancreas. Endocr Pathol (2014) 25(2):181–5. doi: 10.1007/s12022-014-9317-8 24718881

